# Suppression of Subpixel Jitter in Resonant Scanning Systems With Phase-locked Sampling

**DOI:** 10.1109/TMI.2024.3358191

**Published:** 2024-06-03

**Authors:** Vincent D. Ching-Roa, Chi Z. Huang, Michael G. Giacomelli

**Affiliations:** Department of Biomedical Engineering, University of Rochester, Rochester, NY 14627, USA

**Keywords:** Fluorescence microscopy, high-speed imaging, jitter suppression, laser scanning microscopy, resonant scanner

## Abstract

Resonant scanning is critical to high speed and *in vivo* imaging in many applications of laser scanning microscopy. However, resonant scanning suffers from well-known image artifacts due to scanner jitter, limiting adoption of high-speed imaging technologies. Here, we introduce a real-time, inexpensive and all electrical method to suppress jitter more than an order of magnitude below the diffraction limit that can be applied to most existing microscope systems with no software changes. By phase-locking imaging to the resonant scanner period, we demonstrate an 86% reduction in pixel jitter, a 15% improvement in point spread function with resonant scanning and show that this approach enables two widely used models of resonant scanners to achieve comparable accuracy to galvanometer scanners running two orders of magnitude slower. Finally, we demonstrate the versatility of this method by retrofitting a commercial two photon microscope and show that this approach enables significant quantitative and qualitative improvements in biological imaging.

## I. INTRODUCTION

RESONANT scanners are widely used in many different types of microscopy including confocal [1], [2] and two photon [3]–[6] microscopy as well as super-resolution imaging using stimulated emission depletion [7]–[10] microscopy. The combination of wide scan angle and very high imaging rate has driven recent advances in real-time live animal imaging [4], [11], [12] and neuroscience [3], [13], [14], enabled adaptive optics scanning laser ophthalmoscopy [15], [16], two photon excitation ophthalmoscopy [17] and fluorescent lifetime ophthalmoscopy[18] in awake patients as well as accelerated real-time diagnosis of human cancers [19]–[21]. Resonant scanning has also enabled in vivo super-resolution imaging of living animals[10], providing high speed imaging of the subcellular dynamics of live neurons. However, the increasing use of resonant scanning to image transient events, to super-resolve subwavelength features, and for diagnostic medical imaging has brought new attention to the limited temporal and spatial accuracy of most resonant scanning systems stemming from scanning instability and position tracking inaccuracies. These limitations in accuracy hinder new microscopy techniques by corrupting high spatial frequencies, obscuring temporal dynamics and complicating quantitative analysis [2], [22].

Accurate resonant scanning is difficult because, unlike non-oscillatory scanners, which use closed-loop control to precisely position the mirror, resonant scanners are free-running and continually drift in frequency. Thus, the location of the mirror at each point in time cannot be directly controlled, and instead must be inferred from potentially uncertain measurements, resulting in pixels that jitter about their true location. As the resolution and imaging rate of microscopy techniques increases, the uncertainty in scanner position imposes a proportionally more severe effect. Earlier approaches to compensate for jitter focused on optically tracking the mirror position with photodiodes [23] and/or splitting the scanned beam between two microscopes, one of which imaged a known grating, from which the true position of the mirror at each point in time could be inferred [23], [24]. More recently, various computational approaches based on cross-correlation of sequential lines to estimate gross changes in frequency or phase have been described [16], [25], although these approaches are sample-dependent and only partially remove scanner jitter.

The most effective approaches to jitter management have focused on direct measurement of scanner position and then computational correction in post-processing. One straightforward approach deflects a second tracking laser from the mirror and then optically tracks the time that the scanner passes a given angle, eliminating uncertainty in the true mirror position induced by the electronics [23][26], [27]. Due to the accuracy of this method, many resonant scanners are designed to be reflective on both side of the scanner to facilitate optical measurement [27]. If an additional digitizer channel is used to record the analog waveform generated by the tracking laser using the pixel sampling clock, then synchronization jitter between the sampling and resonant frequencies can be measured and each laser scan resampled to remove this jitter [26].

A related computational approach replaces the optical position sensing with analog read out of the pickup coil inside the resonant scanner. By reading out the position coil once per pixel, the location of scanner can be sampled much more finely than the two times per cycle enabled by a single laser and photodiode [28], although some commercial implementations have also used position sensitive diodes to optically read out position continuously [27]. As with optical tracking, if the same digitizer clock is used to read out the position sensor, then synchronization jitter can be measured and removed by resampling.

While highly effective, these methods have limitations. First, both require substantial additional hardware that must be designed into the microscope such as an additional laser and photodiode or require access to the position sensing coil on the resonant scanner which is not readily accessible in some resonant scanners. This is compounded by the need for an additional digitizer channel, which reduces the number of imaging channels available. Second, both methods operate in post-processing, recording real-time data that is jittered, and then correcting it only in review. While, in principle, correction could be performed rapidly enough for nearly real-time display, this still represents a substantial additional processing step that would be incompatible with existing microscope software.

As a result of these practical limitations, no approach has found widespread acceptance yet, and a recent (2020) Nature Protocols recommended simply not using bidirectional resonant scanning due to unacceptable jitter-induced image artifacts in typical resonant scanning systems [22]. In this work, we show a simple, accurate (<1/15^th^ or <1/30^th^ of diffraction-limit for 8kHz and 12kHz scanners, respectively) and intrinsically real-time jitter suppression method without added optical, mechanical, or computational components. As in related work by Tweed [23] and by Xiao et al [29], we use a phase-locked loop (PLL) circuit to perform synchronization and then further incorporate clock denoising.. This method can be retrofitted onto existing microscope systems by connecting a simple circuit in-line with the resonant scanner and is compatible with most microscope system that can use an external sampling clock. No software changes are required. The ability of a PLL to generate rational-number multiples of an input signal is used to enable scanner-synchronous pixel sampling, to denoise the scanner position feedback, and to track the frequency drift of the scanner in real-time, enabling accurate resonant imaging without post-processing. To demonstrate the effectiveness of this method, we quantify jitter characteristics of two common resonant mirrors and the jitter suppression provided by a PLL. We next evaluate the stability of the resonant scanner in the transverse axis of two commonly used resonant scanner models. We show that there is inherent transverse axis wobble which can be intensified by crosstalk coupling with a galvanometer scanner. We also examine the implications of jitter suppression in imaging, particularly in both artifact suppression and PSF broadening. Finally, we demonstrate substantial real-world improvements in image accuracy by imaging cells on a commercial resonant scanning microscope with and without jitter correction.

## II. Methods

### A. Sources of Jitter and PLL Theory of Operation

Multiple sources of jitter are known to affect resonant scanning imaging which we group into three main categories: 1) mirror jitter, 2) feedback jitter, and 3) synchronization jitter. Mirror jitter is attributed to the mechanical instability of the mirror due to physical factors such as inertial effects [30], [31], vibrations [31], or thermal drift [31]. Feedback jitter is due to the limitation on how accurately the scanner position can be tracked electronically and has traditionally been addressed by tracking the mirror position optically [23][24]. Finally, synchronization jitter is the bounded ± 0.5 sample jitter when the resonant scanner and the imaging sampling clock are unsynchronized [26], [28].

A phased locked loop, or PLL, measures the phase difference between a reference input and a variable oscillator. The difference signal feeds back to the oscillator in order to minimize the error. If the feedback loop further integrates a counter and low pass filter, the internal oscillator frequency can be set to a multiple of the averaged reference frequency. So-called ‘zero-delay’ PLLs further measure their own phase shift and incorporate this into the feedback loop, enabling output with exactly zero phase shift. Thus, a zero-delay PLL enables both reduction of the feedback jitter and the generation of higher frequency sampling clocks from kilohertz resonant scan rates without changing the timing seen by a microscope which suppresses synchronization jitter. All experiments were performed with the Si5345 PLL (Skyworks) using the Si5345-EVB evaluation board.

### B. Scanner Jitter and PLL Characterization

**Fig. 1.**
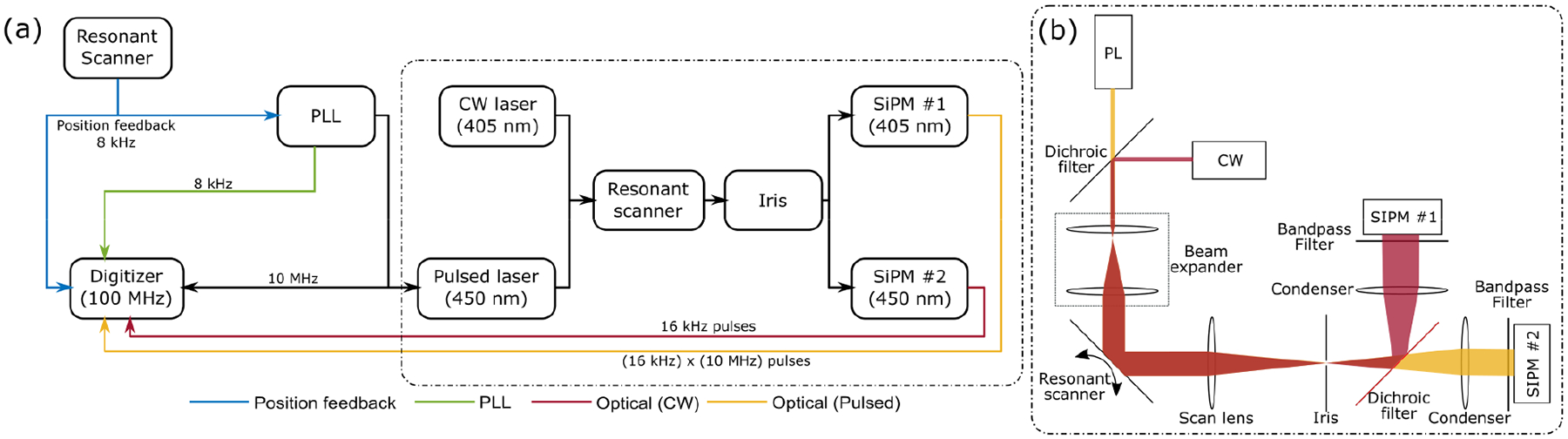
Experimental setup (a) for scanner jitter measurement and the corresponding optical setup (b).

An 8 kHz resonant scanner (CRS 8k, Cambridge Technology) from a commercial galvo-resonant scan-head was evaluated with the slow-axis galvo mirror (6210H, Cambridge Technology) set statically at its zero-voltage position. The system schematic in Fig. 1 illustrates the setup used to characterize the jitter between the scan mirror and the resonant driver clock, as well as the effects of PLL denoising. A continuous wave 405 nm laser (S1FC405, Thorlabs) and a pulsed 450 nm laser (NPL45B, Thorlabs) were scanned with the resonant scanner at its maximum optical scan range (±10°). The scanned beams were focused by the scan lens and an iris was placed at the scan lens’ focal plane. Beams passing through the iris were collected by respective silicon photomultiplier (SiPM) detectors [32], [33] with detected pulses indicating temporal location of the resonant mirror center position. The position feedback signal, which is a TTL signal with rising edge at the scanner turnaround point, was recorded, and also served as a reference input signal for a zero-delay PLL (Si5345, Skyworks). The PLL used a 200 Hz loop bandwidth and generated a denoised 8 kHz clock (x1) and a phase-locked 10 MHz clock (x1250) which triggered the pulsed laser. The digitizer (ATS9440, AlazarTech) captured the following at 100 MHz sampling rate: 1) the resonant scanner position feedback signal, 2) the PLL filtered position feedback signal, 3) the PLL 10 MHz output, 4) the optical signal from the continuous wave laser, and 5) the optical signal from the pulsed laser.

To quantify subsample jitter, waveforms were up-sampled and either rising edges at half amplitude or pulse peaks were located with subsample accuracy to indicate cycle start locations. Since multiple pulses fit into the iris during a scan, the first peak closest to the rising edge was picked for every cycle.

### C. 2D Scan Path Characterization

**Fig. 2.**
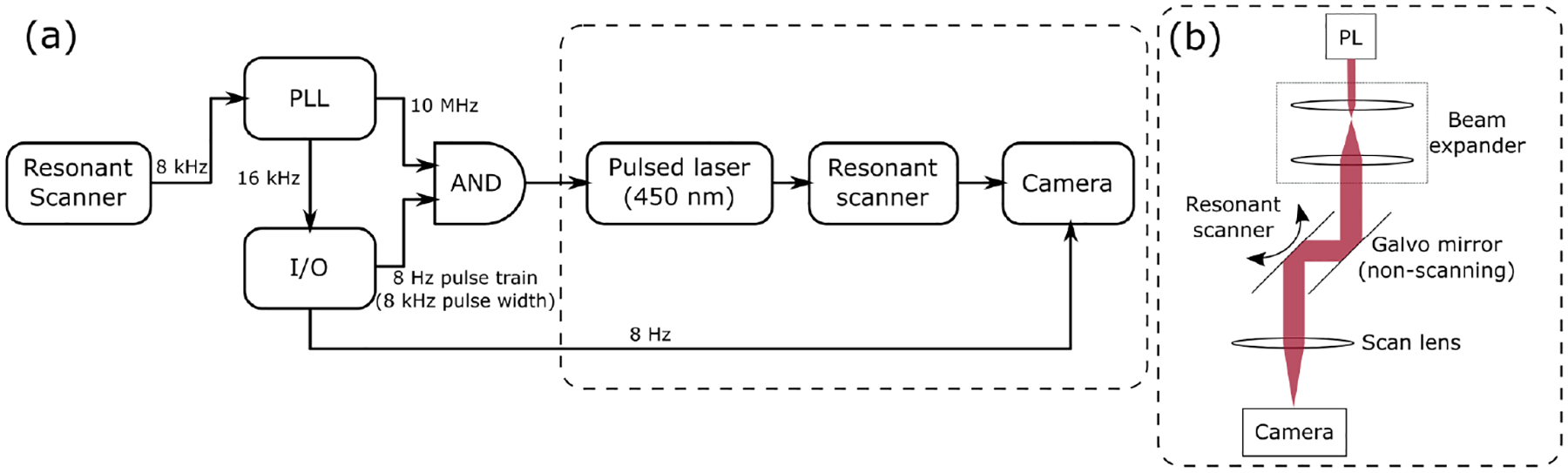
Experimental setup (a) for 2D scanner path characterization with the corresponding optical setup (b)

For quantifying scanner wobble and the spatiotemporal scanning jitter of the scanner, a CMOS camera (BFS-U3-200S6M-C, FLIR) was placed at the focal plane of the scan lens while the same 450 nm pulsed laser was scanned by the resonant scanner at its maximum scan range. The position feedback signal was fed into the PLL running with 200 Hz loop bandwidth which generated a 16 kHz (x2) and 10 MHz (x 1250) clock. The 16 kHz clock went into an I/O card (PCIe-6361, National Instruments) to generate an 8 Hz camera frame trigger. The 8 Hz frame trigger was AND-gated with the 10 MHz clock used to trigger the pulsed laser to ensure that each frame acquired only one forward-backward sweep of the scanner. This experiment was performed for both the 8 kHz resonant scanner and the 12 kHz resonant scanner (CRS 8K, 12K, Novanta Inc.). The experiment setup is summarized in Fig. 2.

The camera only captured the central ~65% of the maximum scan pattern (±10°) of the 8 kHz scanner. Each pulse in the video was spatiotemporally located through up-sampling and subpixel peak-fitting. For the 12 kHz scanner, the full scan pattern was captured but only ~65% of the scan pattern was used for peak-fitting due to overlap between adjacent pulses at the edges.

### D. Phantom and Tissue Imaging

To measure jitter suppression, a two-photon microscope [5], [33] which uses the CRS 12kHz scanner was used to scan a fixed slit in front of a detector. For these measurements, the PLL loop bandwidth was reduced to 100 Hz based on the measurements in Section 3A. The PLL generated a denoised 12 kHz (x1, averaging ~45 scanner periods) and an 80.4 MHz (x6700) clock. Either the position feedback signal or the PLL-generated 12 kHz clock served as the line trigger into the digitizer (ATS9440, AlazarTech). To examine the effects of sampling synchronization, the digitizer was clocked with either the 80.4 MHz clock from the PLL or a free-running 80.4 MHz clock as would be the case if sampling synchronous to a mode-locked laser. Line trigger edges were localized by subsample fitting the center of the slit of the corresponding scan line. An alternative method for line trigger edge localization was also performed as shown in Fig. 6(m) where the forward and backward lines of a scan cycle were up-sampled and cross-correlated to measure the misalignment in bidirectional scanning. Additionally, the effects of jitter on PSF broadening in averaged frames were investigated by averaging subsequent scan lines and quantifying the full-width at half-maximum (FWHM) of the image of the slit.

The experiment above was repeated for better jitter estimation with the CRS 8 kHz using fluorescent powder. Fluorescent powder was imaged with the microscope in its strip-scanning mode with the galvanometer held fixed [5] with the stage velocity such that the slow-axis was more than 5x Nyquist sampled. Imaging was performed for all four combinations of two line-trigger options and two sampling clock options. Again, two-way delay is quantified by subpixel cross-correlating forward-backward line pairs.

For cell imaging, nuclei were stained with SYBR Green and imaged with and without PLL jitter suppression with a retrofitted Bergamo two-photon microscope (Thorlabs Inc.) by enabling the manufacturer’s external clock feature and connecting the PLL. A 1040 nm, 100 fs, 100 MHz laser (YLMO-2W, Menlo Systems) was scanned across a 400 μm field-of-view with 2048-pixel lines after dewarping [34] at 80.4 MHz (12,000 Hz, x 6700) sampling rate. The diffraction-limited resolution with the 20x 0.7 NA objective used is approximately 550 nm. A region at the center of field of view was analyzed to minimize sinusoidal non-linearity of samples, while dewarping was provided by the Thorlabs FPGA hardware.

## III. Results

### A. Resonant Scanner Stability

**Fig. 3.**
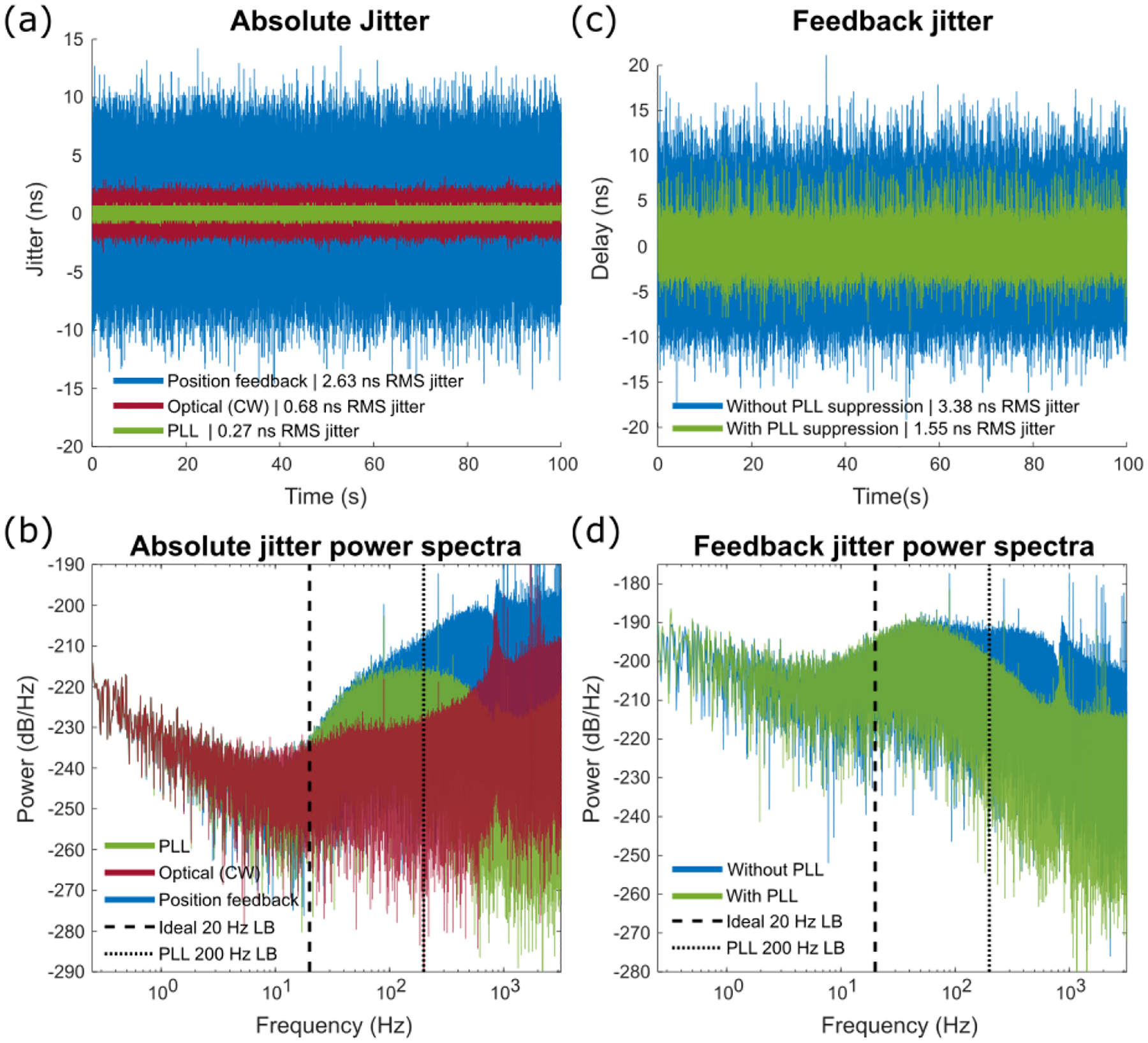
(a) Absolute jitter of the electronic position feedback and mirror position measured optically with a CW laser over 100 seconds from the CRS 8kHz using the PLL-clocked digitizer. (b) Power spectral density of the jitter shown in (a) where dashed and dotted lines mark 20 Hz and 200 Hz respectively. (c) The error between the electronic position feedback signal and the optically measured mirror position with no PLL jitter suppression and with PLL jitter suppression. (d) Power spectral density of the feedback jitter from (c) showing a significant reduction in jitter above 200 Hz.

We first assessed the scan stability of the resonant scanner (CRS 8kHz) and the ability of the PLL to stabilize frequency by tracking the resonant trajectory optically. Feedback jitter (edge-to-edge timing jitter) for the electronic and optical signals were measured based on the subsample localization of the signal rising edge. As shown in Fig. 3(a), the absolute jitter of the position feedback signal from the resonant scanner driver was ~ 4 times larger than optically tracking the mirror position with a continuous wave laser (CW) signal. The position feedback was denoised using the PLL at 200 Hz loop bandwidth which yielded a very low jitter signal in Fig. 3(a). Fig. 3(b) shows the presence of additional broadband electrical noise on top of the true mirror frequency drift, which becomes large above approximately 20 Hz, suggesting that further attenuation of this broadband signal through a lower PLL loop bandwidth should yield a better estimation of the true mirror motion. The power spectra also suggests that a lower loop bandwidth around 20 Hz may further improve stability, as the PLL output with 200 Hz loop bandwidth retained a noise band that is not reflected by the optical signal.

To measure the unsuppressed feedback jitter and the effects of PLL jitter suppression, we plotted the timing jitter of both the position feedback signal and the PLL output relative to the optical signal edges in Fig. 3(c). The effect of PLL denoising is apparent in significant reduction of feedback jitter compared to the unsuppressed configuration with RMS jitter summarized in Table I. Finally, the power spectra of the feedback jitter (Fig. 3(d)) show heavy attenuation of jitter at frequencies above the loop bandwidth. However, unfiltered noise in between 20 Hz and the 200 Hz loop bandwidth remains and contributes to the ~72% of the total time domain jitter in Fig. 3(c).

### B. Scanner Wobble and Slow-axis Crosstalk

Having demonstrated that the PLL could be locked to the true mirror period with high accuracy, we next set out to measure the mirror position within individual scan cycles and assess how uniformly the scanner moves. Using the pulsed laser triggered by the PLL (0.17 ns relative jitter), we recorded the resonant scanner position at 100 ns intervals over individual scans.

**Fig. 4.**
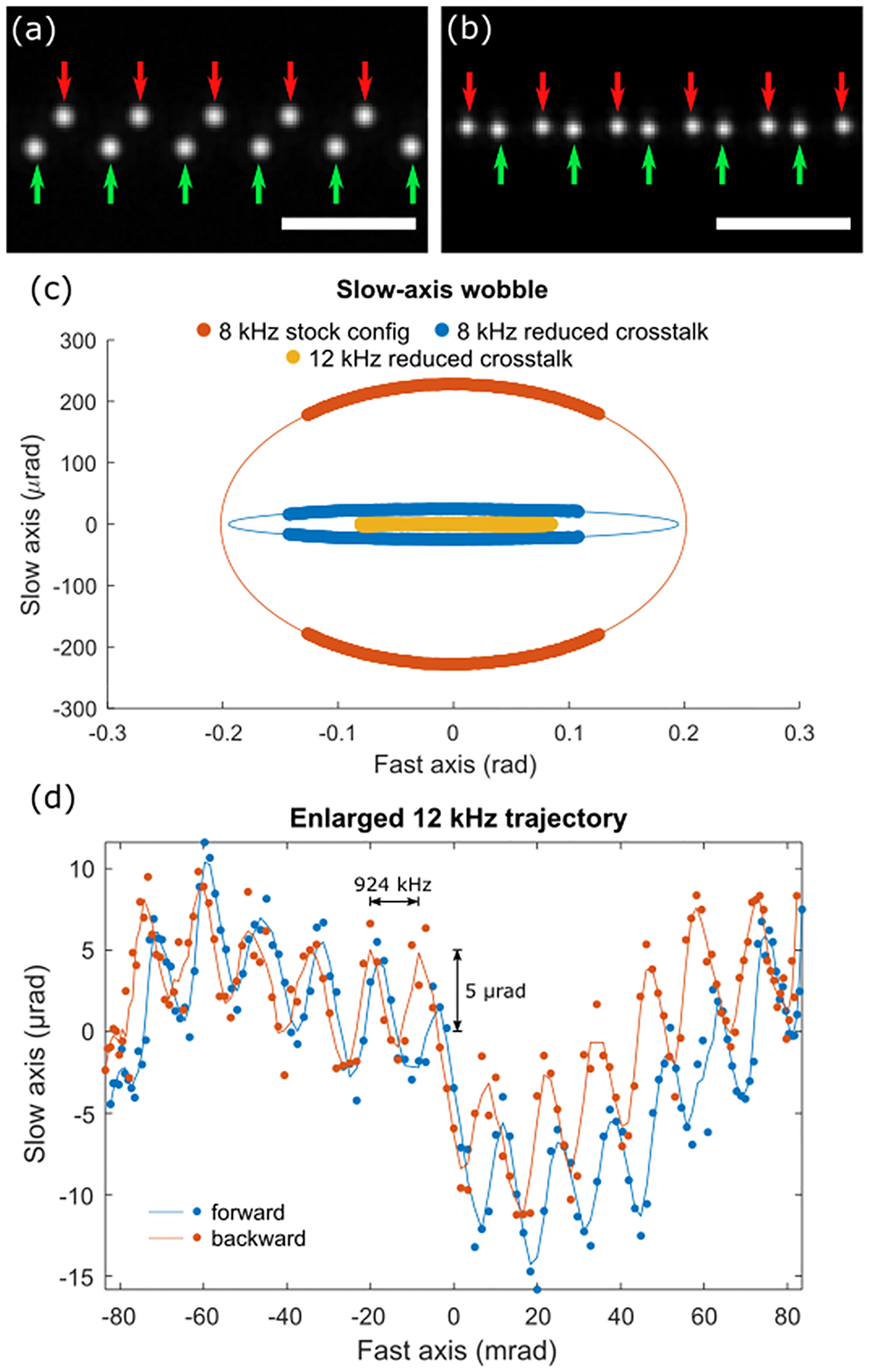
(a) Zoomed in region of a scan cycle of a 10 MHz pulsed laser with CRS 8kHz stock configuration with significant galvo crosstalk. (b) Scan cycle (forward and backward) of a 10 MHz pulsed laser with CRS 8kHz configuration with galvo crosstalk minimized. (c) Recorded scanner trajectory and projected elliptical path fitting of CRS 8kHz scanner with stock and reduced crosstalk configuration and of CRS 12kHz scanner with reduced cross configuration. (d) Representative scanner trajectory of the CRS 12 kHz scanner in reduced crosstalk configuration. The cubic variation common to both directions is due to tiny residual lens distortion. Line-traces show filtered trajectory to suppress stochastic jitter for visualization.

Representative forward-backward sweeps of the resonant scanner (CRS 8kHz) are shown in Fig 4(a–b) for two different cable configurations. To capture the deterministic wobble of the scanner, scan patterns were recorded at 8 fps for 25 seconds and averaged (Fig. 4(c)). From the average scanner trajectories, we observe significant scanner wobble from galvo-resonant drive signal crosstalk. In what is shown as the ‘stock’ configuration in Fig. 4(c), the galvo and resonant drive signals shared a common connector (but different and well-shielded cables). Due to the very high sensitivity to interference of the slow-axis controller (MicroMax 671, Novanta Inc.), crosstalk between drive signals caused the galvo mirror to oscillate synchronously with the resonant scanner, resulting in an elliptical path with maximum wobble of ~ 400 μrad (0.09% of the fast axis angle). Once both drive signals were spatially isolated from each other by adding a second connector, there was a dramatic reduction in wobble down to less than 50 μrad (0.01%). Similarly, we evaluated the scanner wobble for the scan head with CRS 12kHz and found no significant elliptical slow-axis wobble. Instead, the dominant deterministic wobble came from a ~924 kHz oscillation about 5 μrad in amplitude, which may be due to a vibrational mode within the slow axis mirror when mechanically perturbed by the resonant scanner. Fig. 4(d) shows a representative trajectory for the 12 kHz scanner that shows the high frequency oscillation across the slow axis. This remained in-phase over many seconds suggesting that it is generated by the 12 kHz scanner.

**Fig. 5.**
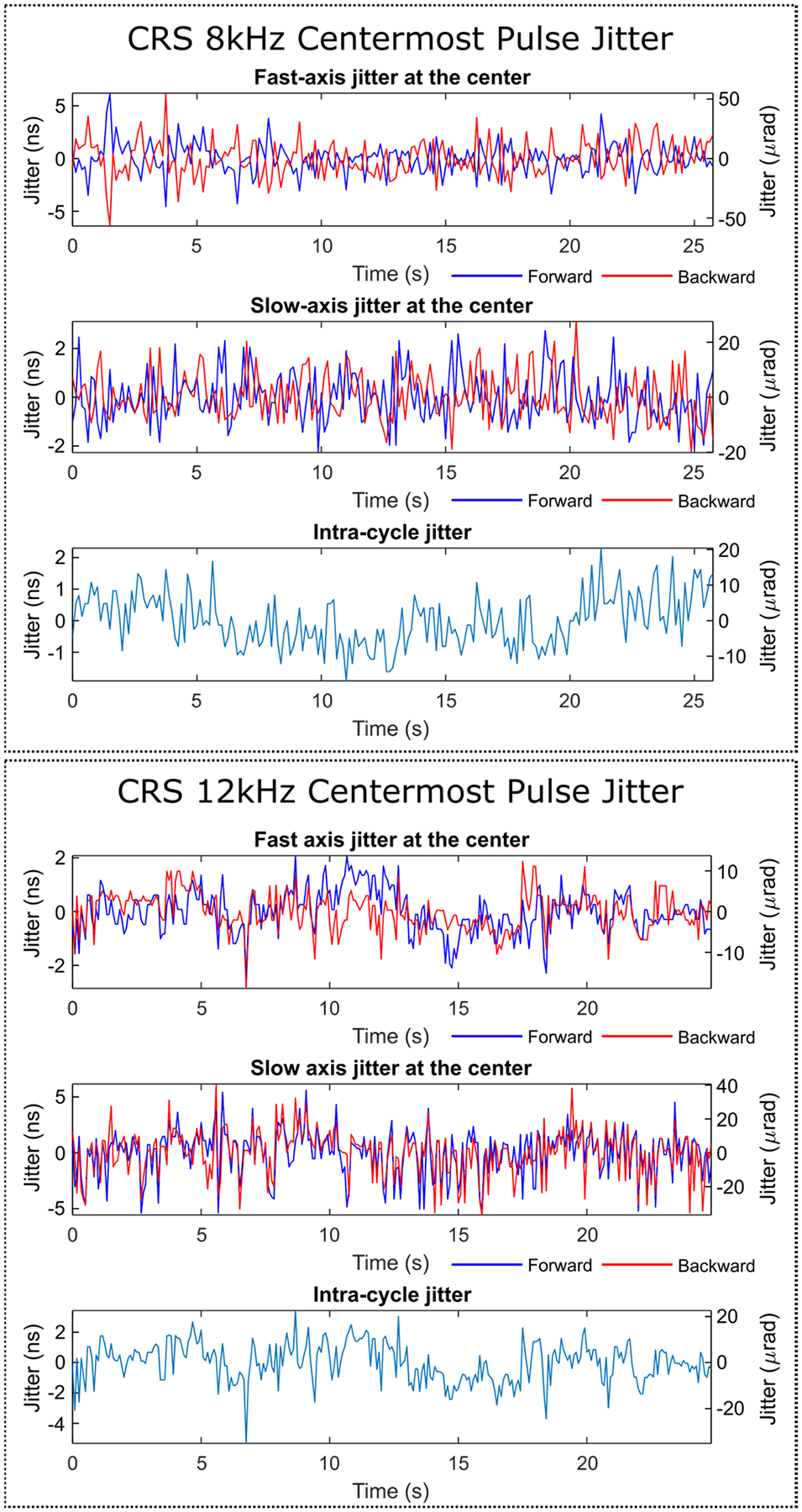
(a-b) Fast axis and slow axis jitter of the centermost pulse of the CRS 8kHz scanner with reduced crosstalk configuration. (c) Intra-cycle jitter between the forward and backward fast-axis jitter for CRS 8kHz. (d-e) Fast axis and slow axis jitter of the centermost pulse of CRS 12kHz with reduced crosstalk configuration. (f) Intra-cycle jitter between the forward and backward fast-axis jitter for CRS 12kHz.

To evaluate 2D scanner jitter, the fast and slow axis spatiotemporal jitter of the centermost pulse of each frame was tracked as shown in Fig.5 and summarized in Table II for both CRS 8 kHz and 12 kHz scanners. For the 8 kHz scanner, the fast-axis jitter in Fig. 5(a) was similar to the 1D measurement using the same loop bandwidth. The slow-axis jitter in Fig. 5(b) was around half the magnitude of the measured fast-axis jitter (<10 μrad RMS), more than an order of magnitude smaller than the diffraction limit. Furthermore, the fast-axis jitter for the forward and backward sweeps from Fig. 5(a) mirror each other, demonstrating highly correlated, mirrored jitter. Consequently, the jitter in the time between the center pulse in the forward sweep and the backward sweep, which we refer to as the intra-cycle jitter, shown in Fig.5(c) is less than 10 μrad RMS. Similarly, the 12 kHz scanner showed extremely low fast axis jitter (Fig.5(d), <6 μrad RMS). However, the forward and backward fast axis jitter do not mirror each other but appear to be positively correlated. This suggests that an external source of jitter dominates (e.g., vibration) although the total intra-cycle jitter (Fig. 5(f)) is negligible. Unlike the 8 kHz scanner, the 12 kHz scanner had a slow-axis jitter (Fig 5(e)) that is higher than its fast-axis jitter. This is in part due to the oscillating wobble for the 12 kHz scanner. However, the slow-axis jitter remains low (<15 μrad RMS, 10x below the diffraction limit). Thus, provided that the crosstalk in the cabling was suppressed, both wobble and intra-cycle jitter were extremely small and would most likely be negligible.

### C. Jitter Suppression During Imaging

In the previous sections, we characterized feedback and mirror jitter with subsample accuracy. However, all imaging systems with a sampling or pixel clock that is not synchronized to the fast axis scanner are subject to additional synchronization jitter due to the random phase differences between the scanner and pixel clock which we show in this section to be the most significant contributor of jitter.

**Fig. 6.**
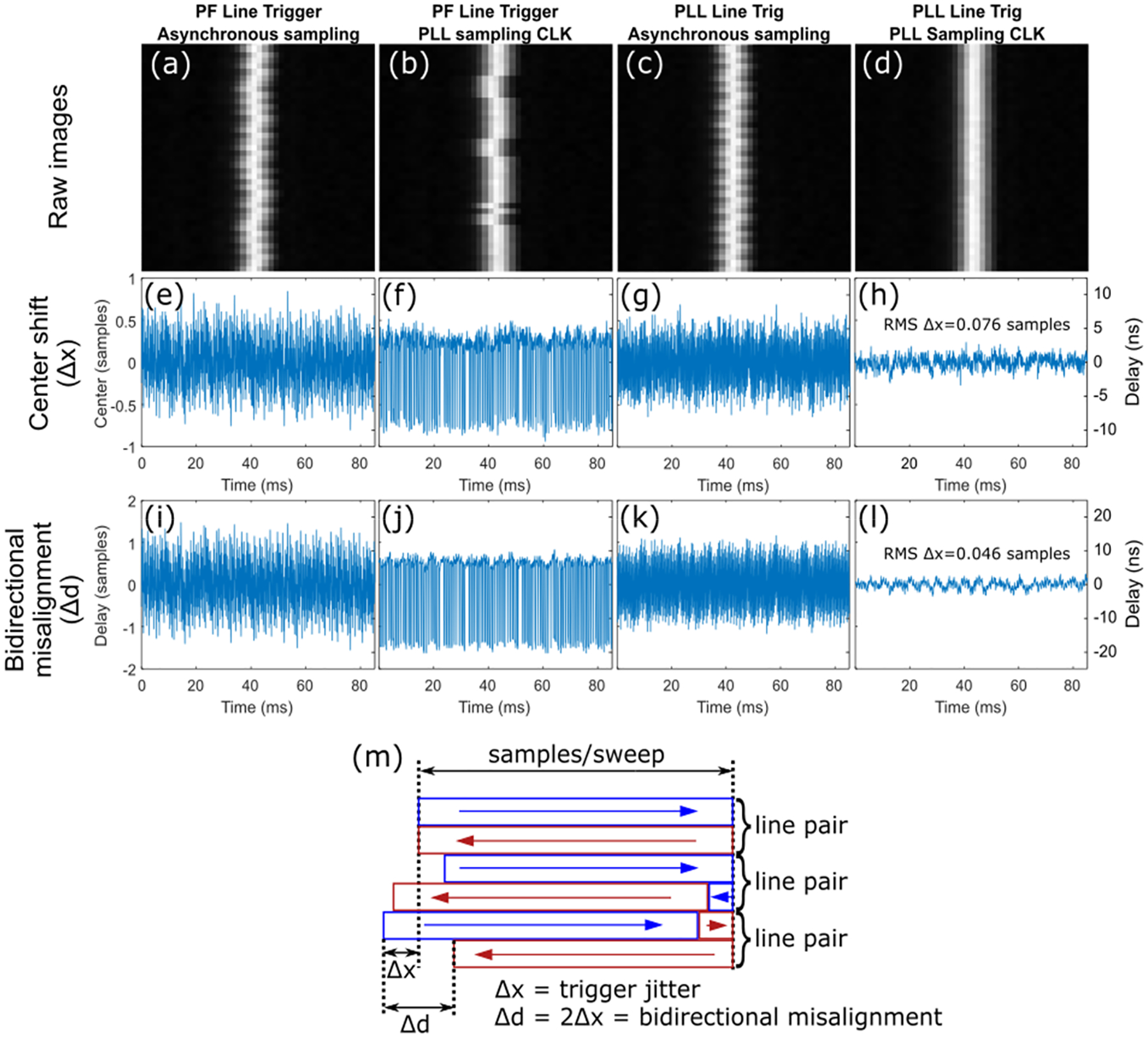
Imaging jitter characterization with CRS 12kHz. (a-d) Fixed slit images with four different configurations of the systems with and without PLL correction for either line trigger, sampling clocks or both. (e-f) Subsample localization of slit center over time for configurations in (a-d) respectively. (i-l) Bidirectional misalignment in forward and backward sweeps of the slit images for configurations in (a-d) respectively. (m) The relationship between trigger jitter and bidirectional misalignment given fixed number of samples per sweep.

To characterize total jitter in an imaging system and the effects of PLL jitter suppression, we imaged a fixed slit (Fig. 6(a–d)) using a microscope with a CRS 12kHz scanner. We then localized the mirror position for each scan cycle through subsample fitting of the peak intensity of the forward-scanned peak (Fig. 6(e–h)). Imaging was done on four system configurations which examine the effects of both PLL line trigger and sampling clock generation. The PLL loop bandwidth was reduced to 100 Hz for all configurations which is the lowest supported bandwidth for our PLL, based off the results in Fig. 2.

While subsample fitting of peaks allowed for reasonable subpixel estimation of imaging jitter, peak fitting is sensitive to noise. We introduce an alternative method for jitter characterization that takes advantage of the information in the backward scan, making it less prone to noise. Because we assign a fixed number of samples for the forward and backward scan lines as demonstrated in Fig. 6(m), jitter (Δx) from the forward scan overflows into the backward scan resulting in a bidirectional misalignment (Δd) that is twice Δx. Such bidirectional misalignment can be calculated by cross correlating the forward and backward scans and finding the shift of maximum correlation. In addition to noise resilience, bidirectional misalignment also provides a more direct metric for observed image jitter in bidirectional imaging. Fig. 6(i–l) shows more accurate estimation of jitter using the bidirectional method. This is particularly pronounced when comparing the extraordinarily low jitter in Fig. 6(h) and Fig. 6(l) (0.076 samples vs 0.046 samples RMS, respectively) which is difficult to measure otherwise.

**Fig. 7.**
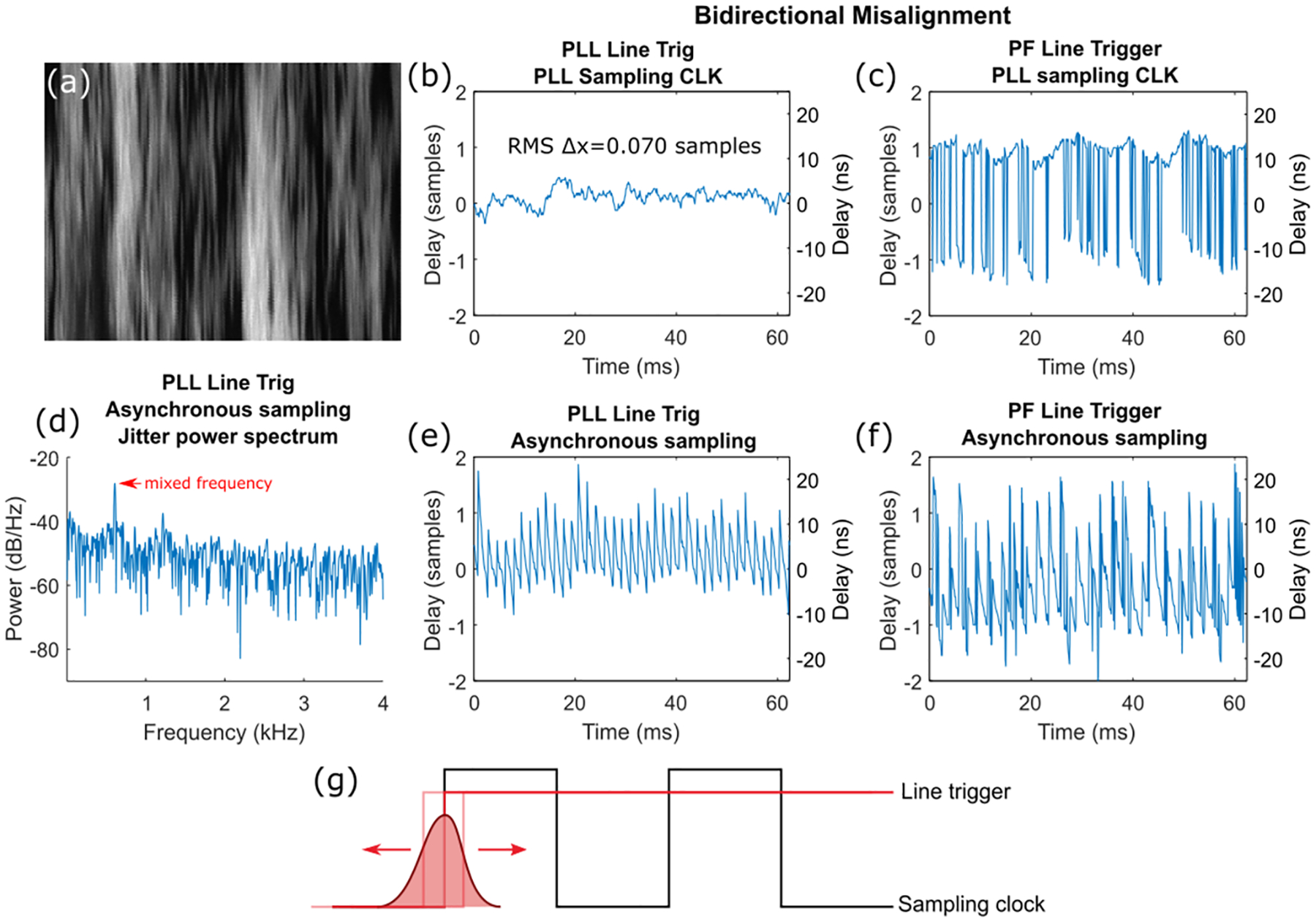
Imaging jitter characterization with CRS 8kHz. (a) Slow-axis oversampled image of fluorescent powder without any PLL jitter suppression. (b,c,e,f) Bidirectional misalignment in forward and backward sweeps of fluorescent powder images from four system configurations with and without PLL correction for either line trigger, sampling clock, or both. (d) Power spectral density of jitter in (e) with a red arrow pointing to the mixed frequency between line trigger and sampling clock. (g) Visualization of the off-by-one trigger jitter effect caused by line trigger jitter. Solid red signal represents ideal line trigger edge while jitter with a probability distribution shown as a Gaussian curve is centered around the ideal edge.

To further explore these measurements, we acquired images of fluorescent powder. These images were oversampled by a factor of >5 across the slow-axis to ensure high correlation between adjacent fast-axis lines. We repeated the bidirectional measurements done in Fig. 6(i–l) for the CRS 8kHz scanner (Fig. 7) using these images.

With more sensitive measurements (Fig. 7), effects of synchronization jitter become more apparent. Relative jitter between the line trigger and sampling clock resulted in two effects. First, feedback jitter results in a distribution of line trigger timings that can fall on either side of the sampling clock (Fig. 7(g)). As a result, part of the distribution past the current clock edge triggers at the next sampling clock edge which results in one-sample timing errors [26]. As shown in Fig. 3(a), the position feedback signal has a large electronic jitter which, combined with slow drift in resonant frequency, continuously pushes the signal back and forth across the threshold. This effect was therefore more dominant in Fig. 7(c) when the noisy position feedback signal was used as the line trigger. Second, if the line and sampling clocks are not synchronized, they cannot have an exact integer multiple frequency relationship and thus will produce a non-zero beat frequency [28]. This leads to an oscillatory jitter due to the mixing of the two frequencies (Fig. 7(d,e)). Without any PLL jitter suppression, both of these effects were superimposed onto each other as seen in Fig. 7(f). Finally, phase-locking both clocks using the PLL removes both effects.

PLL jitter suppression for the 8 kHz scanner resulted in 0.88 ns RMS jitter, fractionally lower than previously measured in Table I due to a lower 100 Hz loop bandwidth and the improved measurement sensitivity from cross-correlation.

Table III summarizes total jitter in imaging microscopes for both slit imaging with CRS 12kHz and fluorescent powder imaging with CRS 8kHz scanners. We provide jitter in both samples and time, as well as in pixels after sinusoidal dewarping at the center of the field.

### D. PSF Broadening with Frame Averaging

**Fig. 8.**
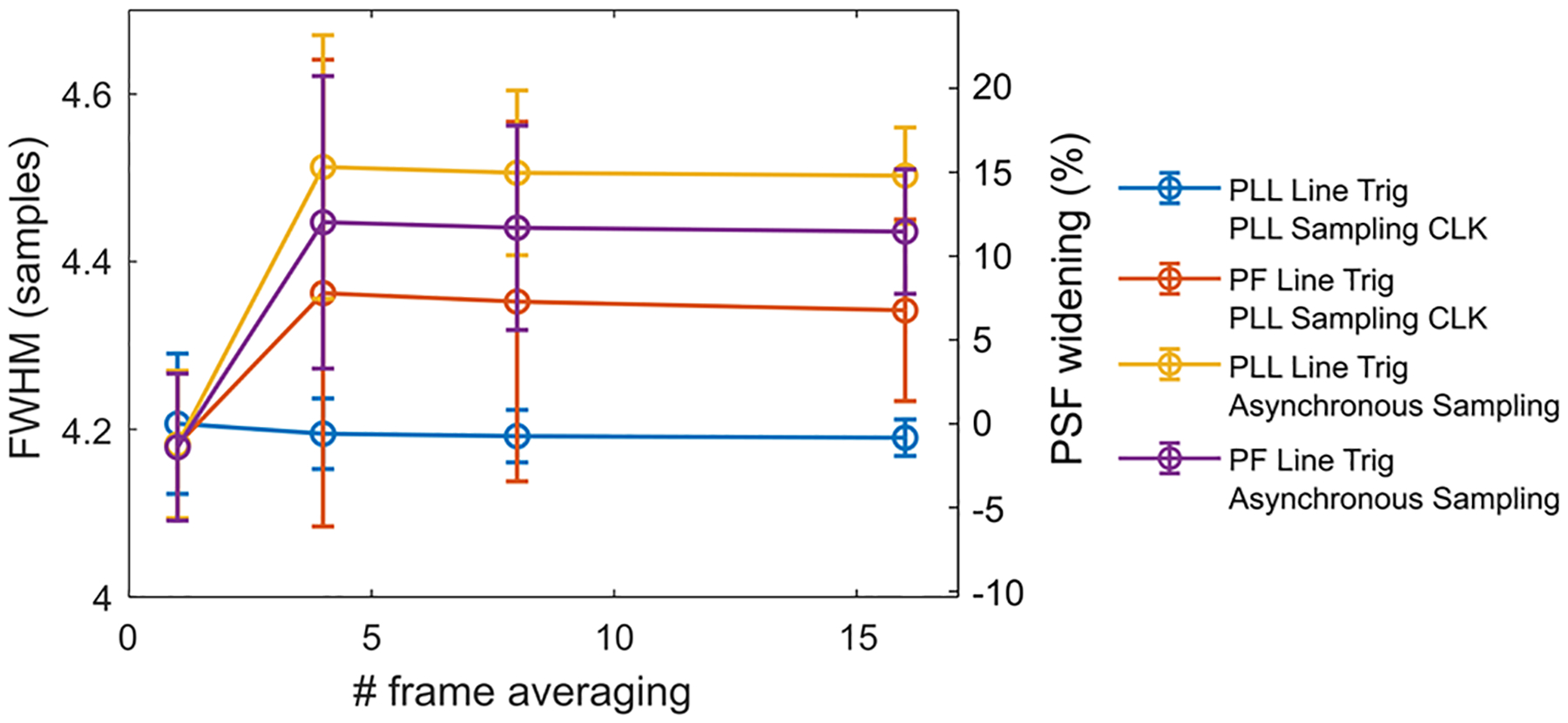
(Left axis) FWHM of the fixed slit images from four system configurations with and without PLL correction for either line trigger, sampling clock, or both with the CRS 12kHz. (Right axis) Percent increase in width relative to diffraction-limited PSF, calculated as the number of pixels increase in width divided by the PSF FWHM in pixels.

While jitter artifacts in single shot images affect position localization, jitter artifacts with frame averaging manifest as PSF broadening as pixels from different locations are combined. Fig. 8 shows the effect of frame averaging on the fixed slit used in Fig. 6. With full PLL jitter suppression, the slit FWHM stayed nearly at the same level with minor improvements due to increasing effective SNR from frame averaging. However, without synchronization, we measured a FWHM widening of up to 0.32 samples at 4 frames averaging. This is equivalent to ~15% of the diffraction limited PSF assuming the full scan range, full aperture, and air immersion with Nyquist-sampled pixels after sinusoidal dewarping.

### E. Improvement in Biological Imaging

**Fig. 9.**
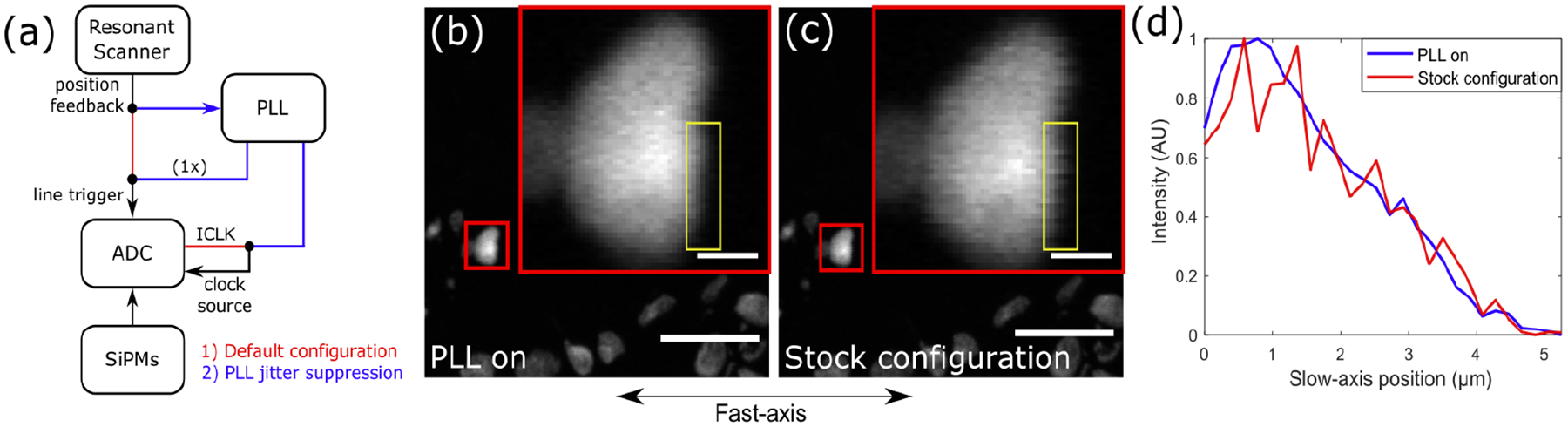
(a) Default microscopy digitizer configuration (red) and with PLL module added for jitter suppression. Nuclei imaging with (b) full PLL jitter suppression and (c) without jitter suppression with a zoomed image inlay enclosed in red. Scale bars: 25 μm (frame), 2.5 μm (inlay). (d) Line profiles in the yellow boxes in (a) and (b).

We retrofitted a Bergamo two-photon microscope (Thorlabs, Inc.) based on the CRS 12kHz resonant scanner. The PLL serves as an add-on module onto the resonant scanner driver clock as shown in Fig 9(a) which is already used for line-triggering and is therefore readily accessible. The PLL provides a clean phase-locked line trigger and sampling clock from which the latter can easily be used as a clock source for the system’s digitizer. Fig. 9(c–d) shows two-photon images of fluorescently stained nuclei with PLL jitter suppression and in the vendor-supplied configuration. The non-jitter suppressed imaged noticeably has stochastic spikes across the slow-axis due to fast-axis jitter while the jitter suppressed image shows a smooth nuclear boundary as seen in Fig. 9(d).

## IV. Discussion and Summary

Resonant scanning systems frequently suffer from artifacts and poor image quality[2], [22]. Conventional wisdom has attributed poor image quality with resonant scanning to instability or accuracy limitations inherent in resonant scanning. Our characterization of two different models of resonant scanner indicates that this is not the case, in agreement with other work [26]. Using two-dimensional, time-resolved trajectory measurements, we show that both resonant scanners have high accuracy along the scan axis, while transverse scanning accuracy is mainly hampered by potential deterministic slow-axis wobble with miniscule non-deterministic slow-axis jitter. Such slow-axis wobble could be minimized through mechanical and electrical isolation between galvo and resonant scanners. Using optical measurements of the mirror trajectory and imaging of small particles, we confirm that the largest source of inaccuracy in resonant scanning systems is synchronization jitter between the resonant and sampling clocks, followed by feedback jitter in the driver electronics. With our entirely passive PLL correction, we were able to suppress both synchronization jitter and feedback jitter from the driver down to the picosecond-level.

Recent papers have characterized resonant scanners and then proposed computational methods to address artifacts and restore full resolution in post-processing [26], [28]. The core feature of these approaches is direct analog measurement of the scanner position using the same digitizer used to record pixel intensity values. Because analog timing information is acquired using the same sampling clock as the pixel data, the scanner position can be determined with subpixel accuracy, and then subpixel shifts applied to each pixel in the image to compensate numerically for the uncertainty in scanner position at time of acquisition. Conceptually, this approach has several advantages, including the possibility of measuring the scanner position multiple times per fast axis sweep. Furthermore, since processing is acausal, the estimation of scanner frequency can incorporate both past and future cycle periods. Collectively, these advantages could potentially enable more accurate measurement of scanner position. However, these post-processing offline methods have the significant downside of requiring additional positional data to be acquired along with pixel data. This typically requires either optical tracking hardware or access to the analog feedback coil to record the analog position data and sacrifices a potential spectral channel from the digitizer. These methods additionally impose substantial computational complexity to compensate for uncertainty in pixel location. In theory, this post-processing correction could be incorporated into resonant sinusoidal dewarping which already requires interpolation. However, this would require that subpixel fitting of the analog position data be performed before dewarping, which is challenging without imposing large delays that make real-time visualization difficult. For most imaging applications, the cost of additional hardware and computational complexities for scanner correction is not a practical tradeoff.

In comparison to previous methods, the approach we perform here requires no computation or modification of software and is intrinsically real-time. The use of a zero-delay PLL reconstructs the current instantaneous frequency from a running average of previous cycles in real-time, and then uses it to construct a sampling clock with picosecond timing error. Fig. 6 shows the striking effect of this improvement in long term accuracy, with the PLL stabilized slit showing as a featureless vertical line, while the un-stabilized slit shows as a fuzzy, meandering line. For both scanners, PLL jitter suppression was able to minimize fast-axis jitter down to less than 600–900 ps, the lowest value reported in the literature. Finally, because the PLL has zero delay and is invisible to software and hardware, in principle most existing microscope with an external clock feature (as is common on two photon microscopes that sample synchronously to a laser) could be retrofitted to improve image quality, as we demonstrated by retrofitting an existing microscope system in Fig. 9.

Because a PLL generates new clocks from historical scanner cycles, the loop bandwidth used to average those cycles represents a key parameter determining the absolute accuracy of the sampling clock. Too low of a loop bandwidth will result in a sampling clock that fails to track changes in the scanner frequency, while too high a bandwidth will result in a noisy estimate of the instantaneous frequency. Initially we selected a bandwidth of 200 Hz (the fastest supported by the Si3545) in order to better track scanner drift. However, optical measurements of the true mirror position (Fig. 3 and Fig. 5) suggested that this was too high, and so we performed subsequent imaging measurements at 100 Hz (lowest supported loop bandwidth), which consistently gave modestly lower jitter, consistent with other data suggesting that scanner frequency changes only very gradually. We speculate based on the jitter power spectrum data in Fig. 3 that an alternative PLL with an even lower loop bandwidth may give further increases in accuracy. Another PLL such as AD9544 (Analog Devices), with a lower minimum input frequency, loop bandwidth and overall cost may be more appropriate than the Si3545 evaluated here. However, our data (Table III) showed with the 12 kHz scanner that uncertainty was less than 1/30^th^ of the angular resolution of the scanner or about 1 pixel error in a 52,000-pixel line, thus further improvements, while possible, may not be practically significant.

A more subtle but no less significant advantage of the approach here is the stabilizing effect the PLL has on the alignment between forward and backward scans in bidirectional scanning. As visualized in Fig. 6(m), most microscopes trigger once per cycle and divide the acquired samples into half for the forward sweep and half for the backwards sweep. As a result, drift in the scanner period results in drift in the number of samples before the scanner turns around, which causes part of the forward sweep to bleed into the backward sweep. This results in a high frequency interlacing artifact if the shift between forward and backward lines is not continuously adjusted during real-time operation. Conversely, with the PLL clocking the sampling clock to the scanner frequency, changes in scanner period are canceled out by changes in the sampling clock period, thus resulting in constant alignment between the forward and backward lines.

While applicable to most resonant scanning microscopes, our method is not applicable in scenarios where laser clock synchronization is necessary such as with FLIM and temporal multiplexing techniques [35]–[37]. However, an alternative jitter suppression method using PLL could still be employed where the PLL is integrated into the feedback loop of the resonant scanner, allowing the scanner to be locked onto the laser, as is implemented in the PLD-1S driver from EOPC. However, this is more difficult to implement and system-specific, making it not generalizable and readily integrable to most existing microscopes.

Finally, real-time subpixel jitter suppression enables higher resolution imaging with resonant scanners, which may be particularly important for ophthalmic applications such as two photon imaging [17] the registration SLO images for retinal FLIM [18] where frames are usually averaged due to limited photon budgets. PSF widening up to 15% of the diffraction-limited resolution is avoided with jitter suppression. Moreover, for super-resolution techniques such as STED, jitter artifacts become increasingly larger compared to the system resolution. Thus, improvements in scanning may also be important in these applications.

## Figures and Tables

**Table I:** Relative RMS jitter between clock signals

Signal pairs		Jitter (ns)
Signal 1	Signal 2	
Position feedback	PLL	3.31
**Position feedback**	**Optical (CW)**	**3.38** ^a^
**PLL**	**Optical (CW)**	**1.55** ^b^

a Feedback jitter without PLL suppression

b Feedback jitter with PLL suppression

**Table II:** 2D Long-term jitter for Centermost Pulse (8Hz sampling, 25 seconds, 200 Hz PLL Loop Bandwidth)

	CRS 8kHz Jitter				CRS 12KHz Jitter			
Source	Angle (μrad)	Time (ns)	Nyq. pixels^a^	Rel. diffr. resolution^b^	Angle (μrad)	Time (ns)	Nyq. pixels^a^	Rel. diffr. resolution^b^
Fast-axis forward	12.80	1.444	0.168	0.105	5.143	0.780	0.053	0.105
Fast-axis backward	14.82	1.670	0.195	0.121	4.549	0.690	0.047	0.121
Fast-axis intra-cycle^c^	7.341	0.828	0.097	0.060	8.323	1.262	0.086	0.060
Slow-axis forward	9.239	1.042	0.122	0.076	13.49	2.045	0.139	0.076
Slow-axis backward	8.478	0.957	0.112	0.069	13.43	2.036	0.138	0.069

a Equivalent jitter in Nyquist-sampled pixels for 1040 nm two-photon imaging (2048-pixel line scan)

b Angular jitter divided by the diffraction limited angular resolution of the scanner at 500 nm

c RMS jitter of the time between the forward sweep and backward sweep center pulses

**TABLE III:** Total Fast-Axis Jitter During Imaging (100 Hz PLL Loop Bandwidth)

Configuration		8 kHz (Fluorescent powder) Jitter				12 kHz (Fixed slit) Jitter			
Line trigger source	Sampling clock source	Samples	Time (ns)	Nyq. Pixels^a^	Rel. diffr. resolution^b^	Samples	Time (ns)	Nyq. Pixels^a^	Rel. diffr. resolution^b^
Pos. feedback	Asynchronous	0.388	4.851	0.560	0.324	0.324	4.024	0.272	0.218
Pos. feedback	PLL	0.448	5.596	0.646	0.443	0.443	5.507	0.373	0.298
PLL	Asynchronous	0.220	2.474	0.285	0.180	0.292	3.633	0.246	0.196
PLL	PLL	0.070	0.880	0.102	0.064	0.046	0.571	0.039	0.031

a Equivalent jitter in Nyquist-sampled pixels for 1040 nm two-photon imaging (2048-pixel line scan) dewarped from 6700 samples per scan cycle

b Angular jitter divided by the diffraction limited angular resolution of the scanner at 500 nm
